# Case of Vaccination

**Published:** 1806-07

**Authors:** 


### *To the Editors of the Medical and Physical Journal.*

GENTLEMEN,

ENTERTAINING as I do the highest opinion of the good effects of vaccination, I have endeavoured to extend it in the neighbourhood in which I live as much as possible; and though it is not so well thought of, as from its merits it ought to be, I have had the satisfaction of vaccinating some hundreds, which, as an encouragement, I do gratis. Having a strong desire to obtain every information on this interesting subject, I have taken the liberty of troubling you with the following case, which you will much oblige me by inserting in your valuable Journal, with your sentiments sentiments thereon. As its appearance is so different from any thing I have before met with, I very much wish for your opinion, whether the person is safe from the small-pox? or whether you would recommend a further trial of vaccination?

I vaccinated a girl about 15 years of age with several others, from the same arm, and desired them as usual to come to me on that day week, when, upon examining her arm, I found that a dark coloured scab was formed, not quite so deep as that of mahogany, with a considerable degree of inflammation around it; there was a slight degree of pain and stiffness in the axillary gland, and she informed me that the pustule had dried off two or three days before. Not being quite satisfied, I inoculated her again with nine others on that day, in the other arm, and upon examination the following week, found the appearance and symptoms exactly to correspond with those before described.* All the others on both days had the disease in the usual way.

I am, &c.

*June* 6, 1806. E. L.

* The Editors do not think that these appearances afford a proof of security against the small-pox; and recommend a repetition of vaccination some months hence.

